# Importance of Both Innate Immunity and Acquired Immunity for Rapid Expulsion of *S. venezuelensis*

**DOI:** 10.3389/fimmu.2014.00118

**Published:** 2014-03-19

**Authors:** Koubun Yasuda, Makoto Matsumoto, Kenji Nakanishi

**Affiliations:** ^1^Department of Immunology and Medical Zoology, Hyogo College of Medicine, Nishinomiya, Japan

**Keywords:** intestinal nematode, Th2 cell, ILC2, IgE, mast cell, eosinophils, IL-33, chitin

## Abstract

In the first part of this review, we described the relevant roles of endogenous IL-33 for accumulation of ILC2 and eosinophils even in the lungs of Rag2^−/−^ mice. Type II alveolar epithelial (ATII) cells express IL-33 in their nucleus and infection with *Strongyloides venezuelensis* induces IL-33 production by increasing the number of ATII cells possibly by the action of chitin. IL-33 from ATII cells induces ILC2 proliferation and at the same time activates them to produce IL-5 and IL-13, which in combination induce lung eosinophilic inflammation, aiding to expel infected worms in the lungs. In the second part, we showed that, although AID^−/−^ mice normally develop Th2 cells and intestinal mastocytosis after infection with *S. venezuelensis*, they need adoptive transfers of immune sera from *S. venezuelensis* infected mice to obtain the capacity to promptly expel *S. venezuelensis*. Thus, intestinal nematode infection induces various Th2 immune responses (e.g., Th2 cell, ILC2, goblet cell hyperplasia, intestinal mastocytosis, smooth muscle cell contraction, local and systemic eosinophilia, and high serum level of IgE and IgG1). However, all of them are not necessary for rapid expulsion of intestinal nematodes. Instead, some combinations of Th2 immune responses are essentially required.

When animals are infected with intestinal nematodes, resistant hosts develop Th2 immune responses, which induce IgG1 and IgE production, intestinal mastocytosis, pulmonary eosinophilia (e.g., Loeffler syndrome), and systemic eosinophilia. *Nippostrongylus brasiliensis* is a gut-dwelling nematode. Goblet cell hyperplasia and intestinal smooth muscle contraction, both of which are induced by the action of Th2 cytokines (IL-4 and IL-13), are indispensable for rapid expulsion of *N. brasiliensis* (1, 2). However, B cells and antibody (Ab) production are not needed for this expulsion (3). Thus, host animals expel *N. brasiliensis* in a T cell but not B cell-dependent manner.

*Strongyloides venezuelensis*, a counterpart of human pathogen *Strongyloides stercoralis*, naturally infects rodents and has been used as an experimental intestinal parasite model (4). In contrast to *N. brasiliensis* expulsion, intestinal mastocytosis is indispensable for rapid expulsion of *S. venezuelensis* (5–7). Furthermore, FcRγ-induced mucosal mast cell (MMC) activation is important for rapid expulsion of *S. venezuelensis* (8), suggesting that Ab-dependent MMC activation is essential for rapid expulsion of *S. venezuelensis* from intestine.

In the life cycle of *S. venezuelensis*, third stage larvae (L3) migrate to the lung, where they induce severe inflammatory change (mouse Loeffler syndrome), characterized by severe eosinophilic infiltration and goblet cell hyperplasia. Then, they leave lungs, migrate to oral cavity, and go down to small intestine, where they become adult worms and induce severe intestinal mastocytosis. Host animals try to expel them by the action of mast cells in intestine. Therefore, there are *two inflammatory sites*; one in the *lung* and the other in the *intestine*. We speculated that these two sites are important for protection against intestinal nematode. Here, we show the importance of innate immunity and acquired immunity for rapid expulsion of *S. venezuelensis*.

## Is Loeffler Syndrome a Protective Immune Response?

We first examined the mechanism how *S. venezuelensis* infection induces pulmonary eosinophilia. Loeffler syndrome is severe pulmonary eosinophilia, and parasite-infected patients often develop this syndrome (9). However, we still do not know why only lungs develop such severe eosinophilic inflammation after infection with intestinal nematodes, such as round worms, hook worms, and *Strongyloides* spp. (9). To understand this mechanism, we used *S. venezuelensis* infected-animal model. As intranasal administration of IL-33 induces severe pulmonary eosinophilia and goblet cell hyperplasia in the lungs of animals (10), we speculated that *S. venezuelensis* infection induces Loeffler syndrome in an IL-33-dependent manner (11).

IL-33 is a member of IL-1 family cytokine (12), stored in the nucleus of cells (13), released when cells are damaged (14), and binds to ST2 (IL-1RL1) on Th2 cells and various types of innate immune cells including mast cells, basophils, eosinophils, and group 2 innate lymphoid cells (ILC2s) (10, 15–19). In the first part of this review, we demonstrate that worms increase IL-33 expression in the lung, which in turn not only induces the accumulation of ILC2s in the lung but also stimulates them to produce IL-5 and IL-13, which in combination induce pulmonary eosinophilia.

## *S. venezuelensis* Infection Failed to Induce Lung Eosinophilia in *IL33*^−/−^ Mice

**Figure 1:**
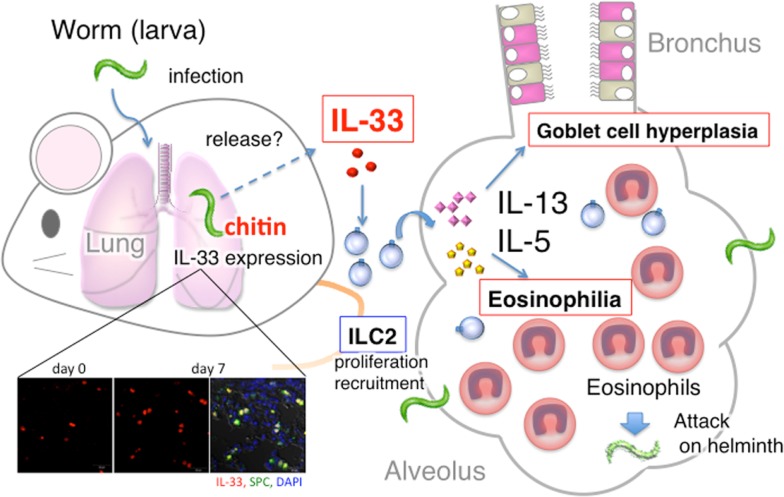
**Summary of host protective pulmonary eosinophilia**. *S. venezuelensis* infection of *Rag2*^−/−^ mice induced severe eosinophilic inflammation, goblet cell hyperplasia, and accumulation of ILC2, and increased the number of IL-33 producing type II alveolar epithelial (ATII) cells. First, IL-33 from ATII cells induced and activated ILC2 to produce IL-5 and IL-13. Then, IL-5 and IL-13 in combination induced severe pulmonary eosinophilia. And, perhaps, these eosinophils, increase their capacity to kill helminth after stimulation with IL-33.

Wild type (WT) C57BL/6 mice, infected with *S. venezuelensis*, developed pulmonary eosinophilia and lung goblet cell hyperplasia at days 5 and 7 after infection. In contrast, *S. venezuelensis* infected IL-33^−/−^ mice failed to develop these changes. These results strongly indicated that *S. venezuelensis* infection induced lung eosinophilic infiltration and goblet cell hyperplasia by induction of IL-33. Therefore, we next tried to determine what type of cells express IL-33. We detected IL-33-expressing cells even before infection. Their number increased and peaked at day 7. We could determine these IL-33-expressing cells as type II alveolar epithelial (ATII) cells, because they are positive for ATII cell marker Pro-Surfactant protein C (Figure 1). Other investigators also reported that influenza virus infection induces IL-33 expression in alveolar epithelial and endothelial cells (20). Influenza virus infection also induces IL-33 expression in the alveolar macrophages (21). However, we could not detect IL-33 expression in F4.80^+^ macrophages in the lungs, suggesting selective activation of ATII cells by *S. venezuelensis*.

## Intranasal Administration of Chitin Induces IL-33 in the Lung

Chitin is a component of the outer membrane of parasites (22), and intranasal administration of chitin beads induces eosinophilic accumulation in the lungs by the action of macrophage and leukotriene B_4_ (23). Thus, we speculated *S. venezuelensis* infection induced pulmonary eosinophila by the action of chitin. We administered chitin into WT mice and IL-33^−/−^ mice, and found that this treatment increased the number of IL-33-expressing ATII cells and IL-33 protein level in the BALF of WT mice. Expectedly, only WT mice developed pulmonary eosinophilia after chitin treatment, suggesting that *S. venezuelensis* infection induces pulmonary eosinophilia at least by the action of chitin to induce an increase in the number of IL-33-expressing ATII cells.

## Induction of ILC2 in the Lungs by *S. venezuelensis* Infection

We next examined whether *S. venezuelensis* infection induces pulmonary eosinophilia without help from Th2 cells. Thus, we infected WT and Rag2^−/−^ mice with *S. venezuelensis*. Both types of mice after infection almost equally increased the number of IL-33-expressing ATII cells and developed lung eosinophilia, indicating that acquired immune cells are dispensable for IL-33-induced eosinophil accumulation in the lungs. IL-33 is a potent inducer of IL-5 and IL-13, which are strongly related with the accumulation of eosinophils (24). Th2 cell is a candidate for the source of these cytokines, however, as we showed previously (10), intranasal administration of IL-33 induces pulmonary eosinophilia even in Rag2^−/−^ mice, excluding contribution of Th2 cells to lung eosinophilic inflammation. ILC2 is another candidate for Th2 cytokine-producing cell in response to IL-33 (25). Therefore, we examined whether *S. venezuelensis* infection induced ILC2s in the lung. We found that *S. venezuelensis* infection induced ILC2s in the lungs of Rag2^−/−^ mice. ILC2s in the BALF started to increase at least at day 7 and increased even beyond day 10. Compared to WT mice, ST2 deficient mice showed little induction of ILC2s. IL-33^−/−^ mice also showed very modest increase of ILC2s. And, administration of IL-33 strikingly increased this proportion (11, 26).

## Pulmonary Eosinophila is Involved in Host Defense Against *S. venezuelensis* Infection

Consistent with the above results, IL-33^−/−^ mice developed modest eosinophilia in their lungs, but they became to develop severe pulmonary eosinophilia after IL-33 treatment. Thus, we examined the contribution of IL-33-induced eosinophilia to worm expulsion. We measured their egg deposition. Compared to IL-33^−/−^ mice, WT mice significantly reduced their egg deposition at day 8. Importantly, IL-33^−/−^ mice could reduce their egg deposition in response to IL-33 treatment. Interestingly, they more rapidly reduced egg deposition than PBS-treated WT mice. These results are consistent with previous reports that eosinophils are required for the rapid expulsion of larvae, which is demonstrated by anti-IL-5 Ab-treated mice and IL-5 transgenic mice (27, 28). The importance of eosinophils in nematode protection was also suggested by a functional study of leukotriene B4 in *S. venezuelensis* infected mice. Numbers of adult worms and eggs/g/feces were greater in 5-lipoxygenase^-/-^ mice or in WT mice treated with leukotriene synthesis inhibitor (MK886) than that in their WT control mice or in PBS-treated WT mice, respectively (29).

Taken together, worms induced IL-33 in the lung by the action of chitin. IL-33 from ATII cells induces and activates ILC2s to produce IL-5 and IL-13, which in combination induce pulmonary eosinophilia and possibly kill helminth (Figure 1). Thus, ILC2s in the lung play an important role in host defense against helminth.

## Does IgE Act in Concert with IgG to Expel *S. venezuelensis*?

Th2 cells induced goblet cell hyperplasia and smooth muscle contraction are essential for rapid expulsion of *N. brasiliensis* (1, 2). However, B cells and antibodies are not required for this expulsion (3). In contrast, Ab-dependent MMC activation is essential for rapid expulsion of *S. venezuelensis* from intestine (8). Thus, host deploys a subset of immune response to expel intestinal nematode, which differs depending on the nature of helminth. As Th2 cells induce various immune responses (e.g., goblet cell hyperplasia, intestinal mastocytosis, local and systemic eosinophilia, and high production of IgG1 and IgE) in mice infected with intestinal nematodes, we tried to determine which components of Th2 immune responses are essentially required for rapid expulsion of *S. venezuelensis*. Thus, in the last part of this review, we show the importance of acquired immunity for rapid expulsion of *S. venezuelensis* from intestine.

## Relevance of Intestinal Mastocytosis and Abs Against Murine Strongyloidiasis

The relevance of intestinal mastocytosis has been well documented in the host defense against murine Strongyloidiasis (5–7, 30). We previously reported that administration of IL-18 induces intestinal mastocytosis and such IL-18-pretreated mice gain the capacity to strongly expel implanted adult worms (7). We further demonstrated that identically pretreated mast cell-deficient WBB6F1-W/W^v^ failed to acquire this capacity. These results strongly indicate that intestinal mastocytosis is required for rapid expulsion of *S. venezuelensis* (7). However, other investigators suggested that induction of intestinal mastocytosis is not sufficient and that FcRγ-induced MMC activation is essentially required for rapid expulsion of *S. venezuelensis* (8). Abraham’s group demonstrated the contribution of Abs (IgM and IgG) and complement to the protection of mice against larval infection with *S. stercoralis* (31, 32). But, because human pathogen *S. stercoralis* can not grow into adult worms in the mice, the role of Abs against adult worms have not been examined in detail.

## *AID*^−/−^ Mice Develop Th2 Cells but Lack the Capacity to Expel *S. venezuelensis*

To solve these issues, we employed activation-induced cytidine deaminase (AID)-deficient mice devoid of Ig class switching (33). AID^−/−^ mice produce more IgM than do WT mice, but lack IgA, IgG, and IgE (34). Although WT mice showed strong production of IgG1 and IgE following *S. venezuelensis* infection, AID^−/−^ mice were unable to produce IgG1 and IgE. WT mice completed expulsion of *S. venezuelensis* by day 12, whereas AID^−/−^ mice required additional 9 days to do so. IL-4 production *in vivo*, which was detected by the *in vivo* cytokine capture assay (35), did not differ between WT mice and AID^−/−^ mice at day 10 after infection. Adult worms of *S. venezuelensis* found in the small intestines of AID^−/−^ mice outnumbered those of WT mice at day 10 after infection. In contrast, like WT mice, AID^−/−^ mice completed expulsion of *N. brasiliensis* by day 10 after infection, excluding contribution of IgG1 and IgE for expulsion of *N. brasiliensis*. The accumulations of mast cells in AID^−/−^ intestines outnumbered those in WT controls at day 14 after infection. AID^−/−^ mice also produced comparable levels of mouse mast cell protease-1 (mMCP-1) to those by WT mice at day 10 after infection, and produced twice as much as did WT mice at day 14 after infection. These data indicated that although Th2-type immune responses and MMC proliferation occurred normally in AID^−/−^ mice, worm expulsion was retarded compared to WT mice due to their no production of class-switched antibodies.

## FcRγ-Induced MMC Activation is Essentially Required for Rapid Expulsion of *S. venezuelensis*

**Figure 2:**
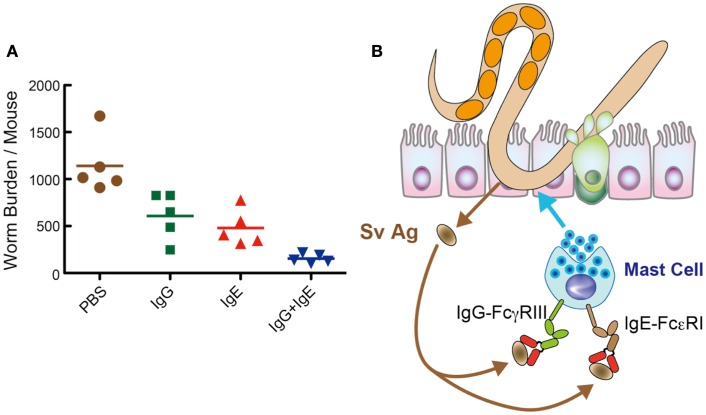
**IgG and IgE collaboratively accelerate expulsion of *S. venezuelensis* infection**. **(A)** To examine which classes of Igs are able to induce worm expulsion. We injected IgG Fr (1.8 mg), IgE Fr (5 μg), or a mixture of IgG Fr (1.8 mg) and IgE Fr (5 μg) into AID^−/−^ mice on day 7 after infection with 4,000 L3, and adult worms were recovered at day 8. As shown here, IgG and IgE reduced worm burdens collaboratively. **(B)** Hypothetical mechanism of IgG- and IgE-mediated worm expulsion.

Thus, we investigated the capacity of immune sera from mice infected with *S. venezuelensis*. AID^−/−^ mice transferred with *S. venezuelensis*-immune sera diminished egg deposition in the feces at day 11, which was 11 days earlier than the normal sera-treated AID^−/−^ mice. Consistently, AID^−/−^ mice transferred with *S. venezuelensis*-immune sera almost completely expelled worms. These effects were due to expulsion of adult worms from the small intestines, but not due to the suppression of fecundity. Because *N. brasiliensis*-immune sera exerted no activities of eliciting worm expulsion, specific Abs against *S. venezuelensis* could play essential roles in expelling worms from the small intestine. Immune sera-derived IgG and IgE induced worm expulsion via Fcγ receptor III (FcγRIII) and Fcε receptor I (FcεRI), respectively. Although FcγRIII^−/−^ mice or FcεRIα^−/−^mice could normally expel *S. venezuelensis*, FcγRIII^−/−^ mice, when their IgE was neutralized by anti-IgE, or FcεRIα^−/−^mice, when their IgG-binding to FcγRIII was blocked by anti-FcγRIII, showed markedly reduced ability to expel *S. venezuelensis*. Additionally, combined administration of IgG and IgE showed a collaborative effect on *S. venezuelensis* expulsion (Figure 2A). These data revealed that IgG and IgE played redundant roles but acted in concert to accelerate *S. venezuelensis* expulsion. IgG or IgE was not able to promote worm expulsion in mast cell-deficient WBB6F1-W/W^v^ mice, indicating that mast cells are cellular targets of IgG and IgE.

In summary, *S. venezuelensis* infection induced IgG and IgE activate FcγRIII and FcεRI, respectively, expressed on mast cells. Then, mast cells get fully activated and expel *S. venezuelensis* promptly from the small intestine (Figure 2B). Recently, El-Malky et al. also reported the importance of B cells in immunity against surgically transferred adult worms of *S. venezuelensis* using B cell-deficient JHD mice (36).

In this review, we showed that acquired lymphoid cells, exemplified by Th2 cells and B cells and innate lymphoid cells, exemplified by ILC2s, are important for rapid expulsion of intestinal nematode. First, IL-33-induced ILC2s induce pulmonary eosinophilia (Loeffler syndrome), which is the lung-based first defense line for *S. venezuelensis*. Second, Th2 cells induce intestinal mastocytosis and Ab production by B cells (e.g., IgG and IgE), which in combination build up the second defense line based on IgG1 plus IgE-mediated MMC activation in the small intestine.

## Conflict of Interest Statement

The authors declare that the research was conducted in the absence of any commercial or financial relationships that could be construed as a potential conflict of interest.
